# Species-specific isotope dilution analysis of monomethylmercury in sediment using GC/ICP-ToF-MS and comparison with ICP-Q-MS and ICP-SF-MS

**DOI:** 10.1007/s00216-021-03497-z

**Published:** 2021-07-23

**Authors:** Sebastian Faßbender, Marcus von der Au, Maren Koenig, Jürgen Pelzer, Christian Piechotta, Jochen Vogl, Björn Meermann

**Affiliations:** 1 Division 1.1 - Inorganic Trace Analysis, Federal Institute for Materials Research and Testing (BAM), Richard-Willstätter-Str. 11, 12489 Berlin, Germany; 2 Gembitzer Str. 23, 13053 Berlin, Germany; 3 Division 1.8 - Environmental Analysis, Federal Institute for Materials Research and Testing (BAM), Richard-Willstätter-Str. 11, 12489 Berlin, Germany

**Keywords:** Mercury speciation, GC/ICP-ToF-MS, Legacy pollution, Finow Canal, Environmental analysis

## Abstract

A recently introduced inductively coupled plasma-time-of-flight-mass spectrometer (ICP-ToF-MS) shows enhanced sensitivity compared to previous developments and superior isotope ratio precision compared to other ToF and commonly used single-collector ICP-MS instruments. Following this fact, an improvement for isotope dilution ICP-MS using the new instrumentation has been reported. This study aimed at investigating whether this improvement also meets the requirements of species-specific isotope dilution using GC/ICP-MS, where short transient signals are recorded. The results of the analysis of monomethylmercury (MMHg) of a sediment reference material show that isotope ratio precision of ICP-MS instruments equipped with quadrupole, sector-field, and time-of-flight mass analyzers is similar within a broad range of peak signal-to-noise ratio when analyzing one isotopic system. The procedural limit of quantification (LOQ) for MMHg, expressed as mass fraction of Hg being present as MMHg, *w*(Hg)_MMHg_, was similar as well for all investigated instruments and ranged between 0.003 and 0.016 μg/kg. Due to the simultaneous detection capability, the ICP-ToF-MS might, however, be more favorable when several isotopic systems are analyzed within one measurement. In a case study, the GC/ICP-ToF-MS coupling was applied for analysis of MMHg in sediments of Finow Canal, a historic German canal heavily polluted with mercury. Mass fractions between 0.180 and 41 μg/kg (*w*(Hg)_MMHg_) for MMHg, and 0.056 and 126 mg/kg (*w*(Hg)_total_) for total mercury were found in sediment samples taken from the canal upstream and downstream of a former chemical plant.

**Graphical abstract:**

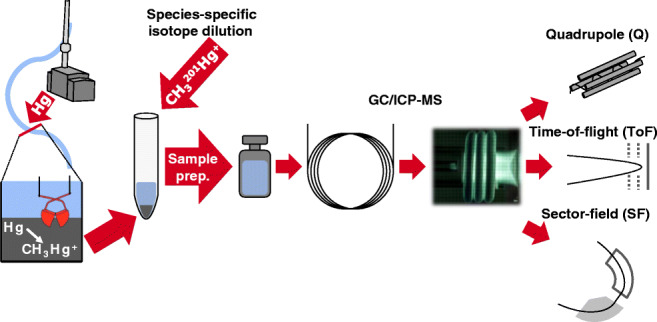

**Supplementary Information:**

The online version contains supplementary material available at 10.1007/s00216-021-03497-z.

## Introduction

Species-specific isotope dilution (SSID) analysis is a primary method producing most reliable and precise results [1, 2]. Isotope ratio precision is the crucial factor associated with the measuring instrument contributing to the measurement uncertainty in SSID analyses, and improvements in isotope ratio precision could translate into higher precision of the results. The latest generation of inductively coupled plasma-time-of-flight-mass spectrometers (ICP-ToF-MS) was shown to exhibit largely improved precision and consistency of isotope ratios over a wide concentration range, as well as improved sensitivity as compared to earlier ICP-ToF-MS systems [3]. Since ICP-ToF-MS instruments work with a quasi-simultaneous detection of all *m*/*z* ratios, they were also shown to be superior to instruments with sequential mass analyzers, such as quadrupole (Q) and single-collector magnetic sector-field (SF) ICP-MS instruments, regarding isotope ratio precision [4, 5]. However, these comparisons have only been conducted with standard continuous liquid introduction analyses and are not readily transferable to the measurement of isotope ratios in (short) transient signals, as it is the case for hyphenations of ICP-MS with separation techniques, such as GC, HPLC, IC, or CE. This is due to poorer statistics resulting from the short time available for the isotope ratio measurement and small analyte quantity [6].

These hyphenations of ICP-MS, especially using HPLC and GC, have substantial advantages in analyzing (trace) elemental species, including high sensitivity and selectivity [7, 8]. Moreover, depending on the matrix, complex sample preparation procedures are necessary and may lead to losses of analyte or species transformation [9], problems that can best be overcome with SSID approaches facilitated by the isotope-specific detection capability of ICP-MS [10]. Studies applying SSID for HPLC/ and GC/ICP-MS analyses have been conducted mostly for speciation of Hg [11–15], Pb [14], S [16], and Sn [13, 14, 17]. In terms of isotope ratio precision, studies applying GC/ICP-ToF-MS for species-specific isotopic analysis have reached relative standard deviations (*s*_rel_) of 0.2–0.5% using high analyte concentrations [18, 19]. For quantification with SSID, a good precision also for low concentrations, i.e., small peaks, is mandatory.

An area of broad interest is Hg speciation because of its complex biogeochemical cycle [20], including natural methylation processes producing extremely toxic monomethylmercury (MMHg), especially in the aquatic environment. Sediments and soils can act as sinks for inorganic Hg species, while they are simultaneously sources of volatile (Hg^0^) and organic species, such as MMHg [20–22], although the amount of substance fraction of MMHg in total Hg is suggested to be as low as 0.1–1% [20, 23]. Natural background levels of Hg in soils and sediments are thought to be in the order of 0.1 mg/kg [20, 22], corresponding to natural MMHg mass fractions between 0.1 and 1 μg/kg. Due to its lipophilicity and strong affinity to thiol groups present in biomolecules containing the amino acid cysteine [24]. MMHg has a high potential for bioaccumulation and biomagnification. This is highlighted by exceptionally high MMHg concentrations found in fish and fish predators [20] as well as aquatic insects [25] and birds [26] living in close vicinity to Hg-contaminated waterbodies. In addition to that, MMHg ingested mainly by consumption of predatory fish and rice can also pass the blood-brain barrier in humans [20].

One example of a highly Hg-contaminated waterbody is Finow Canal, the oldest artificial waterway still in operation in Germany, with Hg mass fractions of up to or higher than 100 mg/kg in the sediment [27, 28], which has been attributed to a chemical plant producing (among others) mercury-based seed dressings [28, 29]. Despite this high mass fraction of Hg, anaerobic conditions, and high amounts of organic matter and iron [28], which are all favorable for Hg methylation [20, 30], no investigations of Hg speciation have been conducted there up to now.

In this study, we evaluate the performance of a recently commercialized ICP-ToF-MS in SSID GC/ICP-MS and compare it with widely used ICP-Q-MS and ICP-SF-MS instruments. The practicability of the ICP-ToF-MS is shown by a case study conducting mercury speciation analysis in sediments of Finow Canal on length from before the known polluted sited into the confluence with Oder-Havel Canal.

## Materials and methods

### Samples

**Fig. 1:**
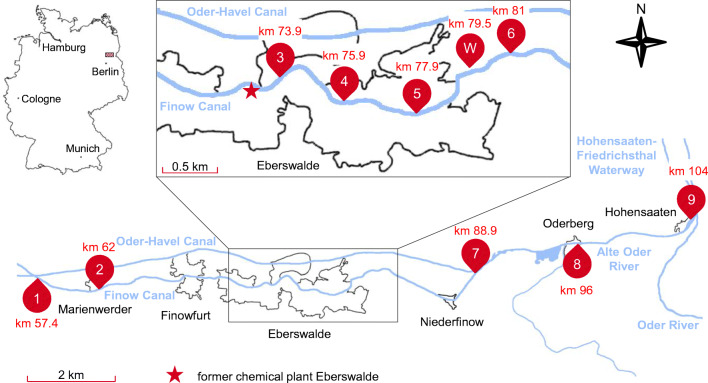
Map of the sampling area at Finow Canal in Brandenburg, Germany, north-east of Berlin with labeling of all sampling sites

Samples were taken from eight locations following the Finow Canal (Brandenburg, Germany) downstream from the crossing of Finow Canal and Oder-Havel Canal near Marienwerder to the confluence of both canals near the town Niederfinow (Fig. 1). Two additional samples were taken from Alte Oder River, ca. 8 and 16 km downstream of Niederfinow, in Oderberg and Hohensaaten, respectively. Sample W was the only non-sediment sample. It was taken from the uppermost centimeters of the soil of a washing bed next to Finow Canal, where sediment removed from the canal bed within the city of Eberswalde has been deposited periodically up to the 1980s [29]. This material was sampled with a stainless-steel shovel after removing the plants covering the soil surface. The sediment samples were taken from piers extending up to 2 m into the canal with a Van Veen grab sampler. At each location, the grab sampler was used several times, so that up to 2 L of sediment material was collected and filled into a glass jar. However, at sites 5 and 8, the sediment layer was much thinner and considerably less amount of sample material was collected. More details on the sampling sites can be found in Table S1 of the Supplementary Information (ESM).

The samples were freeze-dried and sieved to a particle size below 250 μm before analysis. Freeze-drying is a gentle preservation method which was shown to have no significant influence on Hg speciation [31]. In between sampling, freeze-drying, sieving, and analysis, the samples were stored refrigerated at 4 °C. For correction of the residual moisture content, the dry matter fraction of each sample and the reference materials was determined in triplicate according to DIN EN 15934 [32].

### Reagents

Ultrapure water (resistivity ≥18.2 MΩcm) was obtained from a Milli-Q Reference water purification system (Merck Millipore, Darmstadt, Germany). Concentrated nitric acid (HNO_3_) and hydrochloric acid (HCl) (both p.a. quality from Th. Geyer, Renningen, Germany) were further purified by a two-stage sub-boiling distillation (PicoTrace, Bovenden, Germany). Copper sulfate (CuSO_4_) pentahydrate (p.a.), glacial acetic acid (p.a.), methanol (for residue analysis), and sodium hydroxide solution (NaOH, 32%, p.a.) were also purchased from Th. Geyer. Methylmercury chloride (99.8%), ethylmercury chloride (technical), tetrahydrofuran (THF, stabilizer-free), hydrofluoric acid (HF, 48%, ultrapure), and sodium sulfate (p.a., anhydrous) were purchased from Sigma-Aldrich (Taufkirchen, Germany). The derivatization agent sodium tetra-n-propylborate (NaBPr_4_, >98.0%), n-hexane, and dichloromethane (DCM) (both picograde, for residue analysis) were obtained from LGC Standards (Wesel, Germany). Sodium thiosulfate pentahydrate (p.a.) was purchased from Merck (Darmstadt, Germany).

^201^Hg-enriched mercury oxide (HgO) was purchased from Chemotrade (Düsseldorf, Germany) and ^201^Hg-enriched monomethylmercury solution (*w*(MMHg) = 5.1 mg/kg in 3:1 acetic acid/methanol mixture) from ISC Science (Oviedo, Spain). Dilutions of 6.9 μg/kg and 0.068 μg/kg ^201^Hg-enriched MMHg in methanol with dilute HCl (*w* ≈ 0.02 kg/kg) were prepared gravimetrically for spiking the samples. The ^201^Hg-enriched HgO was dissolved in dilute HNO_3_ and diluted further with ultrapure water to a HNO_3_ concentration of 1 mol/L. This stock solution was diluted with HNO_3_ (*c* = 1 mol/L) to a mass fraction of 20.7 mg/kg Hg, which was determined by reverse-ID ICP-MS prior to this study. A second dilution of 1.1 mg/kg in HNO_3_ (*c* = 1 mol/L) was prepared gravimetrically.

For validation of the analytical method, the sediment reference materials IAEA-456 (IAEA, Vienna, Austria; 0.125 μg/kg MMHg as Hg and 0.077 mg/kg total Hg), ERM-CC020 (BAM, Berlin, Germany; 27.4 mg/kg total Hg), and ERM-CC580 (IRMM, Geel, Belgium; 75.5 μg/kg MMHg and 132 mg/kg total Hg) were used. Details on the ^201^Hg-enriched spike and reference materials can be found in Table S2 of the ESM.

### Mercury speciation analysis

#### Extraction

The extraction procedure was based on a procedure described by Carrasco and Vassileva [33] using an acidic extraction solution of CuSO_4_ and HNO_3_ and DCM to directly extract MMHg into the organic phase for separation of inorganic Hg. The required volume of spike solution was added to the sample with the extraction solution and estimated for every sample to target an ^201^Hg/^202^Hg ratio of approximately 1. The actual amount of spike solution added was determined gravimetrically. A Na_2_S_2_O_3_ solution was used to back extract MMHg into the aqueous phase for propylation, and hexane was used for extraction of the propylated Hg species. A detailed description of the sample preparation procedure can be found in section S1 of the ESM.

Very low concentrated sediment samples (IAEA-456 and samples 1 and 8) were prepared with a sample amount of 1 g; of all other samples, an amount of 0.25 g was used. Procedural blanks were prepared by following the whole sample preparation procedure without sediment and with (for determination of LOQ) and without ^201^Hg-enriched MMHg spike. Three replicates were prepared for each sample and reference material per day, with exception of IAEA-456, which was prepared in three replicates on 1 day and only once on each of three other days due to the limited amount of this material. Each replicate was analyzed three times with GC/ICP-ToF-MS, with exception of the replicates of low concentrated samples (IAEA-456 and samples 1, 2, and 8), which were analyzed five times. For measurements with GC/ICP-Q-MS and GC/ICP-SF-MS, two replicates of ERM-CC580 and three procedural blanks with and one procedural blank without ^201^Hg-enriched MMHg spike were prepared. Each replicate of the unspiked blank and the reference material was analyzed three times, and each replicate of the spiked blanks five times.

#### Analysis

The analysis of MMHg was conducted using a Trace 1310 GC (Thermo Fisher Scientific, Bremen, Germany) coupled to an icpTOF 2R (TOFWERK, Thun, Switzerland) via a heated transfer line (Thermo Fisher Scientific, Bremen, Germany) and a dual introduction injector. The straight inlet of the injector was connected to the transfer line, which consists of a sulfinert-coated stainless-steel tubing surrounded with a heating jacket. The GC column was fed into the stainless-steel tubing ensuring that the column end was located in the middle of the heating jacket. An additional gas line was also connected to the transfer line, so that the GC effluent was carried into the plasma within a gas flow of 0.6 L/min argon gas leading to rapid transfer and minimized band broadening. The additional gas was guided through a 3-m coiled stainless-steel tubing located inside the GC oven to allow for pre-heating of the gas. The second inlet of the injector was connected via tygon tubing to a 20-mL glass cyclonic spray chamber equipped with a glass concentric nebulizer introducing a constant flow of a 1 μg/L Tl standard solution in HNO_3_ (*w* ≈ 0.01 kg/kg). This was necessary for two separate reasons. In ICP-MS, instrumental mass fractionation compromises the measured isotope ratio of the sample leading to a bias in results of ID analyses. This mass bias must be corrected for by analyzing a sample with a known isotope ratio (e.g., natural isotopic composition). Due to the drift of plasma conditions, it is preferred to use an internal standard rather than a separate measurement. Because the MMHg spike is only enriched in ^201^Hg but not monoisotopic, Tl was used as an internal standard since it is similar in atomic mass and does not lead to spectral interferences with Hg isotopes. The wet aerosol was also used to introduce oxygen for complete combustion of the organic compounds eluting from the GC column. When working with dry aerosol, incomplete combustion of organic compounds (solvent, analytes) could lead to carbon deposits on the cones of the ICP-MS instrument, especially in splitless injection mode. The GC was equipped with a 30 m TG5-SilMS column (0.25 mm i.d., 0.25 μm film thickness; Thermo Fisher Scientific, Bremen, Germany) and a programmable temperature vaporizing injector (PTV) operated in splitless mode with an injection volume of 2 μL. The injection program used was a heating ramp of 10 °C/s from 250 to 400 °C. Further details on operating parameters can be found in Tables S3 and S4 of the ESM.

For coupling of the GC to an iCAP Q ICP-Q-MS and an Element 2 ICP-SF-MS (both from Thermo Fisher Scientific, Bremen, Germany), the same interface and GC parameters were used.

The data acquisition parameters of the ICP-MS instruments, especially the integration (or dwell) time, were optimized to allow for a maximum signal-to-noise ratio (S/N) and a reasonable number of data points for low S/N. In general, an increasing integration time results in increasing S/N and decreasing number of data points per chromatographic peak. However, for isotope ratio precision, both a high S/N and a high number of data points per peak are favorable. It should be noted that the range of mass fractions of Hg being present as MMHg (*w*(Hg)_MMHg_) of the samples and reference materials was very large, between 0.125 and 75.5 μg/kg, resulting in very small peaks for low mass fractions and prominent peaks for high mass fractions. Therefore, the acquisition parameters could not be optimized for maximum isotope ratio precision, which is also largely dependent on the concentration/peak height of the analyte. This means that the resulting isotope ratio precision is rather a realistic one than a maximum precision attainable with the respective instrument for transient signals. Based on these considerations and tests with MMHg of natural isotope ratio, a compromising integration time of 100 ms per isotope was selected. All other instrumental parameters can be found in Tables S5 and S6 of the ESM.

#### Data evaluation

For consistent data treatment, the data from GC/ICP-MS analyses were evaluated using Microsoft Excel 365. With respect to the simultaneous detection, ICP-ToF-MS is similar to multicollector-ICP-MS (MC-ICP-MS), several approaches for calculating precise isotope ratios from transient MC-ICP-MS signals have been described, and linear regression (LRS) and peak area integration (PAI) methods have been found to be best suited [34, 35]. The LRS approach, however, is not suited for low S/N because it requires data without baseline correction. Therefore, a baseline-corrected PAI approach of a previous work [36] was adapted for the determination of the isotope ratio of ^201^Hg/^202^Hg of the MMHg peak. The average baseline intensity and standard deviation (*s*) were determined from 50 data points each before and after the peak. The average baseline intensity was subtracted from every data point of the peak and peak data points were used for calculation when the intensity was larger than the average baseline intensity plus 3×*s*. The sum of these data points was used as the intensity of the respective isotope. A mass bias correction was performed following the Russell law using a Tl solution supplied by continuous liquid introduction as shown in Eqs. 1 to 3.

1 $$ {R}_{\mathrm{Tl},\mathrm{experimental}}={R}_{\mathrm{Tl},\mathrm{true}}\times {\left(\frac{m_{203\mathrm{Tl}}}{m_{205\mathrm{Tl}}}\right)}^f $$

2 $$ f=\frac{\ln \left(\frac{R_{\mathrm{Tl},\mathrm{experimental}}}{R_{\mathrm{Tl},\mathrm{true}}}\right)}{\ln \left(\frac{m_{203\mathrm{Tl}}}{m_{205\mathrm{Tl}}}\right)} $$

The experimental ^203^Tl/^205^Tl isotope ratio (*R*_Tl,experimental_) was determined by averaging the isotope ratio of every data point within the peak. The isotopic abundances used for calculating the true Tl isotope ratio (*R*_Tl,true_) and isotopic masses of Tl (*m*_203Tl_, *m*_205Tl_) were taken from the Commission on Isotopic Abundances and Atomic Weights (CIAAW) [37–39]. The mass bias factor (*f*) was then applied on the experimental ^201^Hg/^202^Hg isotope ratio (*R*_Hg,experimental_):

3 $$ {R}_{\mathrm{Hg},\mathrm{corr}}=\frac{R_{\mathrm{Hg},\mathrm{experimental}}}{{\left(\frac{m_{201\mathrm{Hg}}}{m_{202\mathrm{Hg}}}\right)}^f} $$

The applicability of this procedure was checked by analyzing the ^201^Hg/^202^Hg ratio of unspiked samples, and the mean corrected ^201^Hg/^202^Hg (*R*_Hg,corr_) was within the uncertainty range of the natural isotope ratio. The mass bias corrected Hg isotope ratios (*R*_Hg,corr_) of repeated measurements of one sample replicate were averaged and subsequently used for calculation of the mass fraction of Hg being present as MMHg (*w*(Hg)_MMHg,sample_) of the replicate as shown in Eq. 4. All sample results were corrected for the dry matter fraction (*DM*).

4 $$ w{\left(\mathrm{Hg}\right)}_{\mathrm{MMHg},\mathrm{sample}}=\frac{M_{\mathrm{Hg}}\times {m}_{\mathrm{spike}}\times w{\left(\mathrm{MMHg}\right)}_{\mathrm{spike}}\times \left({R}_{\mathrm{Hg},\mathrm{corr}}\times {A}_{202\mathrm{Hg},\mathrm{spike}}-{A}_{201\mathrm{Hg},\mathrm{spike}}\right)}{DM\times {m}_{\mathrm{sample}}\times {M}_{\mathrm{MMHg}}\times \left({A}_{201\mathrm{Hg},\mathrm{natural}}-{R}_{\mathrm{Hg},\mathrm{corr}}\times {A}_{202\mathrm{Hg},\mathrm{natural}}\right)} $$

The molar mass of the spike MMHg (*M*_MMHg_) was calculated from the isotopic masses of Hg isotopes taken from CIAAW [38] and abundances given by the manufacturer. The molar mass of Hg (*M*_Hg_) was taken from CIAAW [38], and the mass fraction of the spike dilution (*w*(MMHg)_spike_) was calculated based on the mass fraction of the stock solution given by the manufacturer and the gravimetric dilution. All masses including the mass of spike dilution added to the sample (*m*_spike_) and the mass of the sample (*m*_sample_) were weighed on an analytical balance (RC201D, Sartorius, Göttingen, Germany). Isotopic abundances of the sample (*A*_201Hg,natural_; *A*_202Hg,natural_) were taken from CIAAW [39], and isotopic abundances of the spike (*A*_201Hg,spike_; *A*_202Hg,spike_) were taken from the manufacturer’s information.

The measurement uncertainty of *w*(Hg)_MMHg_ was calculated for each replicate applying the Kragten method [40] for Eq. 4. The standard error of the mean (*s*_*m*_) of the Hg isotope ratio and the dry matter fraction were used as the uncertainty components of the respective quantities. For plotting the results, all *w*(Hg)_MMHg_ of the replicates of a sample were averaged, and a pooled uncertainty (*u*_pooled_) was calculated from the uncertainties of the replicates (*u*_1_, *u*_2_, …, *u*_*n*_) and the number of replicates (*n*) as shown in Eq. 5.

5 $$ {\mathrm{u}}_{\mathrm{pooled}}=\sqrt{\frac{{{\mathrm{u}}_1}^2+{{\mathrm{u}}_2}^2+\dots +{{\mathrm{u}}_{\mathrm{n}}}^2}{\mathrm{n}}} $$

The pooled uncertainty was then combined with the standard error of the mean of the replicates (*s*_m,replicates_) to give the final combined uncertainty (*u*_combined_) of *w*(Hg)_MMHg_ as shown in Eq. 6.

6 $$ {u}_{\mathrm{combined}}=\sqrt{{u_{\mathrm{pooled}}}^2+{s_{\mathrm{m},\mathrm{replicates}}}^2} $$

The expanded uncertainty (*U*) with a coverage of approximately 95% was calculated by multiplying *u*_*pooled*_ with the coverage factor (*k* = 2). As procedure blank samples prepared in parallel to the sediment samples using the whole extraction and derivatization procedure did not show any MMHg peak and spiked procedure blank samples resulted in very low negative mass fractions, a blank correction could be omitted and no cross-contamination was observed.

### Total mercury analysis

#### Digestion

For analysis of total Hg mass fractions, 0.5 g of sample was weighed into a 35-mL quartz microwave vessel fitted with a PFA liner, and the ^201^Hg-enriched spike solution was added. For digestion, 6 mL conc. HNO_3_ (*w* ≈ 0.65 kg/kg), 2 mL conc. HCl (*w* ≈ 0.31 kg/kg), and 3 mL conc. HF (*w* ≈ 0.48 kg/kg) were added. The sample was digested in a CEM Discover SP-D microwave (Kamp-Lintfort, Germany) at 200 °C for 20 min. The supernatant of the resulting digest was taken off and diluted to an average acid concentration of 1–2 mol/L. Hg was separated from the matrix using disposable PP anion exchange columns (DWK Life Sciences, Wertheim, Germany) filled with 1.5 mL AG1-X8 resin (200–400 mesh, Bio-Rad, Feldkirchen, Germany). The columns were washed with elution reagent (0.1 g/kg L-cysteine in 0.02 kg/kg HNO_3_) and 4×5 mL ultrapure water. Conditioning was done with 3 mL conc. HCl, 5 mL ultrapure water, and 5 mL HNO_3_ (*c* = 1 mol/L). Subsequently, the diluted sample digest was loaded onto the column, and the matrix was washed out with 3×5 mL HNO_3_ (*c* = 1 mol/L) and 5 mL ultrapure water. Hg was eluted with 2×5 mL L-cysteine (*w* = 0.1 g/kg) in HNO_3_ (*w* = 0.02 kg/kg). For analysis, the sample solutions were diluted with HNO_3_ (*w* = 0.02 kg/kg) to give an appropriate signal intensity. This matrix separation procedure was necessary to ensure matrix-matching between the samples and the ICP Hg standard solution used for mass bias correction because instrumental mass fractionation is strongly influenced by the sample matrix.

#### Analysis

Total Hg analysis was conducted on an Element 2 sector-field ICP-MS (Thermo Fisher Scientific, Bremen, Germany) equipped with a self-aspirating glass concentric nebulizer (nominal uptake rate 0.2 mL/min) and a glass cyclonic spray chamber (20 mL). The instrumental parameters used can be found in Table S7 of the ESM. Three replicates per sample were prepared, and three measurements were conducted for each replicate. For mass bias correction, an ICP Hg standard solution was analyzed for natural ^201^Hg/^202^Hg. The background Hg mass fraction was determined by analyzing three blank samples spiked with ^201^Hg-enriched Hg solution. The measurement uncertainty was calculated as described in the MMHg section with the background mass fraction as additional component.

## Results and discussion

### Validation of the SSID GC/ICP-ToF-MS and ID ICP-MS analytical methods

The analytical method for the analysis of MMHg was validated by analyzing the reference materials ERM-CC580 and IAEA-456 using the procedures for high and low concentrated samples, respectively. Recovery and repeatability were evaluated by performing analyses of the reference materials on four different days. The repeatability of the isotope ratio of one sample was 0.66% (pooled *s*_rel_ of *m* = 3 repeated measurements of each of the *n* = 12 sample replicates) for ERM-CC580 representing samples with high *w*(Hg)_MMHg_ and 4.7% (*m* = 5, *n* = 6) for IAEA-456 representing samples with low *w*(Hg)_MMHg_. The repeatability of the sample preparation was 1.8% (*s*_rel_ of n = 12 sample replicates) for ERM-CC580 and 4.6% (n = 6) for IAEA-456. The mean recoveries with their associated expanded uncertainties are (108 ± 7) % (*n* = 12) for ERM-CC580 and (105 ± 10) % (*n* = 6) for IAEA-456. The analytical method for the analysis of total Hg was validated by analyzing the reference materials ERM-CC580, ERM-CC020, and IAEA-456 in triplicate. The mean recoveries with their associated expanded uncertainties are (103 ± 3) % for ERM-CC580, (100 ± 3) % for ERM-CC020, and (107 ± 9) % for IAEA-456. The results of all analyses of the reference materials were not significantly different from the certified values as shown by *E*_*n*_-values of < 1, determined according to DIN EN ISO/IEC 17043 [41].

The limits of quantification (LOQ) of the complete analytical procedures were determined by spiking blank samples with the ^201^Hg-enriched spike solutions in triplicate. The LOQ was calculated as 10×*s* of the blank replicates and was 0.008 μg/kg (expressed as *w*(Hg)_MMHg_ for a sample weight of 1 g) in case of MMHg and 0.01 mg/kg (expressed as *w*(Hg)_total_ for a sample weight of 0.5 g) in case of total Hg. The mass fractions of all samples were above the respective LOQ.

### Comparison of time-of-flight-, quadrupole-, and sector-field ICP-MS instruments for SSID GC/ICP-MS

**Fig. 2:**
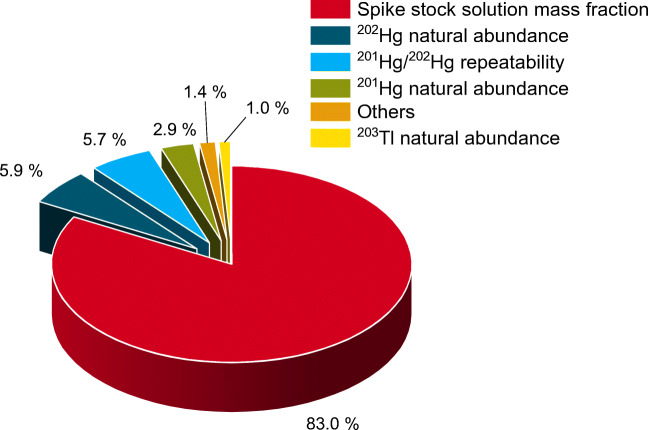
Relative uncertainty contributions for each input quantity to the combined uncertainty, exemplarily shown for the undiluted ERM-CC580 extract analyzed with GC/ICP-ToF-MS (*n* = 3 replicates). Sixteen input quantities with separate contributions of 0.5% or lower are summarized as “Others”

The performance of GC/ICP-ToF-MS, GC/ICP-Q-MS, and GC/ICP-SF-MS for SSID of MMHg was compared based on the analysis of the ERM-CC580 reference material. The mean recoveries with their associated expanded uncertainties are (108 ± 7) % (*n* = 12) determined with GC/ICP-ToF-MS, (109 ± 7) % (*n* = 2) with GC/ICP-Q-MS, and (108 ± 7) % (*n* = 2) with GC/ICP-SF-MS. The results clearly demonstrate the equivalence of the three investigated instruments, not only in terms of recovery but also regarding the uncertainty. However, *w*(Hg)_MMHg_ is very high in ERM-CC580 resulting in high peaks and easy and precise isotope ratio calculation, regardless of the used instrument. An investigation of the uncertainty budget (Fig. 2) revealed that the combined uncertainty of *w*(Hg)_MMHg_ is largely dependent on the uncertainty of the mass fraction of the ^201^Hg-enriched MMHg spike solution and only to a small extent on the uncertainty of the experimental isotope ratio.

**Fig. 3:**
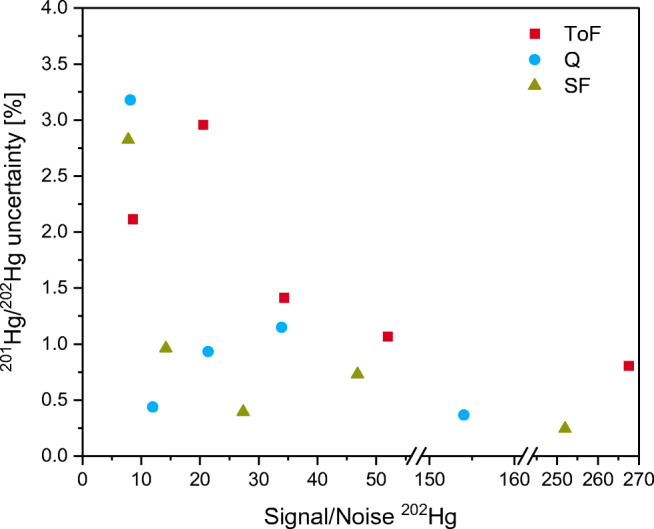
Relative standard uncertainty (*k* = 1) of ^201^Hg/^202^Hg in relation to the signal-to-noise ratio of the ^202^Hg peak of a diluted extract of ERM-CC580. Dilution factors 1 to 175, uncertainty based on *m* = 3 repeated measurements of each sample with the respective GC/ICP-MS coupling using a ToF, Q, and SF instrument. The signal-to-noise ratio is the average of *m* = 3 repeated measurements

For a more thorough investigation in the region of lower concentrations, the hexane extracts of the reference material were diluted by a factor of 10 to 175 with hexane and analyzed again with all three instruments. For this, only one sample replicate was prepared. Quite surprisingly, the results were still similar, with only slightly wider uncertainty ranges for the highest dilution. The precision of the measured ^201^Hg/^202^Hg ratio depends on the S/N of the peaks, as shown in Fig. 3, and decreases with decreasing S/N. No significant differences between the three mass analyzers can be observed, and the higher uncertainty of the ToF at S/N 20.5 may be a random outlier and cannot be conclusively interpreted without more data.

Assuming an infinite precision of the measured ^201^Hg/^202^Hg ratio (the only uncertainty component depending on the sample), the relative combined uncertainty of the resulting *w*(Hg)_MMHg_ is 3%. It becomes clear that above a S/N of 10, the ^201^Hg/^202^Hg ratio precision is below 1.5%, which accounts for less than 25% of the combined uncertainty of *w*(Hg)_MMHg_ or an increase of the combined uncertainty of 1% (to then 4%). Therefore, above S/N 10, the combined uncertainty is dominated by the uncertainty of the spike mass fraction, and small differences in isotope ratio precision are of no consequence. Below S/N 10, the influence of the measured ^201^Hg/^202^Hg ratio on the combined uncertainty increases, but this effect is in the same order of magnitude for all three instruments.

The LOQ of the MMHg analysis for all three instruments was determined as described in the previous section. The resulting LOQ were 0.008 μg/kg, 0.016 μg/kg, and 0.003 μg/kg for ToF, Q, and SF instruments, respectively, calculated for a sample weight of 1 g and expressed as *w*(Hg)_MMHg_. Although there is a spread of factor 5, the differences are most likely not significant in practice and are more probably due to random effects than to instrumental properties.

These results show that although reported for continuous liquid introduction, ICP-ToF-MS is not significantly superior to sequential Q- or SF-based instruments in terms of isotope ratio precision for SSID in transient signals, when one element is analyzed. The superiority of ICP-ToF-MS in measurements using conventional liquid introduction is apparently not readily transferable to transient signals. This is most probably due to the measurement principle of the ToF mass analyzer, which uses pulsed ion packages from the continuous ion stream generated by the ICP ion source [42]. The dwell time for one ion package resulting in a single mass spectrum is 46 μs [43]. Although the detection of ions is (quasi-) simultaneous, the number of ions per ion package is very small, and thereby isotope ratio precision of single spectra is low. By averaging a great number of spectra within a selected integration time, the isotope ratio precision can be increased. However, for the analysis of transient signals, the integration time is limited by the peak width because a certain number of data points per peak (around 20 to 30) is required to maintain an acceptable precision also for small (low and narrow) peaks. Therefore, the superior precision attainable with long integration times of several seconds, as described for continuous introduction [3, 4], cannot be reached when acquiring narrow and low transient signals. It is expectable that this will be different if more than one isotope system is analyzed because additional analytes do not influence ToF data acquisition since they are already detected (simultaneously) anyway. As opposed to this, with Q and SF mass analyzers, every additional analyte will result in less data points per peak or shorter dwell times, and thus significantly decreasing isotope ratio precision. Thus, the strength of ToF might come to light once multi-isotope systems and short transient signals are investigated.

### Mercury speciation analysis of Finow Canal sediment

**Fig. 4:**
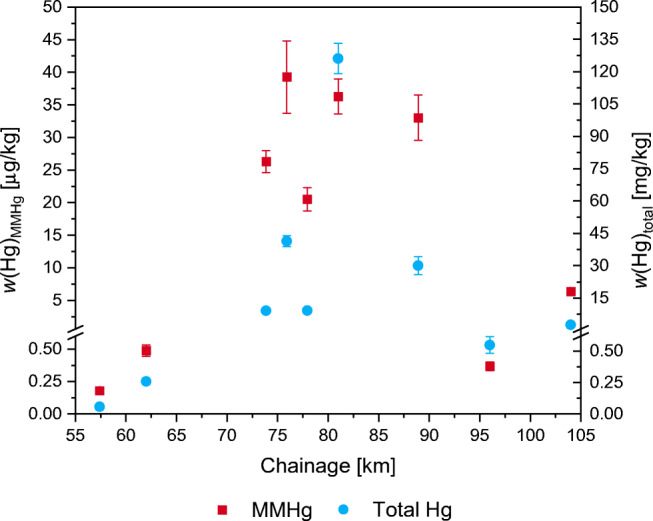
Monomethylmercury (MMHg), *w*(Hg)_MMHg_, and total mercury, *w*(Hg)_total_, mass fractions of sediment samples taken from Finow Canal. Note the different units for MMHg μg/kg and for total Hg mg/kg. All mass fractions are based on dry weight of the < 250 μm grain size fraction. Error bars represent expanded uncertainty *U* (*k* = 2) of *n* = 3 sample replicates and are partially smaller than the symbol size. The exact position of the last two sampling sites was estimated

The SSID GC/ICP-ToF-MS and ID ICP-SF-MS analysis of the sediment samples from Finow Canal revealed *w*(Hg)_MMHg_ between 0.180 and 39 μg/kg, and *w*(Hg)_total_ of 0.056 to 126 mg/kg as shown in Fig. 4. The variation in relative expanded uncertainty (6–14%) is caused by a varying repeatability of the sample preparation (replicates). Because this was not the case for reference materials, which in contrast to the samples are homogenized by grinding, the varying repeatability might be attributable to sample heterogeneity. However, even a relative expanded uncertainty of 14% is still adequate for identifying polluted sites. While *w*(Hg) in samples 1 (km 57.4), 2 (km 62), and 8 (km 96) were below 0.5 μg/kg and 0.6 mg/kg for MMHg and total Hg, respectively, and thereby close to the postulated natural background, samples 3 (km 73.86) to 7 (km 88.91) show strongly elevated levels above 20 μg/kg and 9 mg/kg, respectively. This increase in between sampling sites 2 (km 62) and 3 (km 73.86) corresponds most probably to the former location of a chemical plant that produced mercury-based seed dressings and to the results of total Hg analysis in previous studies [28]. Interestingly, *w*(Hg)_MMHg_ is nearly constant over the course of Finow Canal with a drop after the confluence with the Oder-Havel Canal (sample 8, km 96), but even approximately 16 km downstream of the confluence, a *w*(Hg)_MMHg_ around one order of magnitude above natural background was found (sample 9, km 104). For *w*(Hg)_total_, the maximum value of more than 100 mg/kg agrees with previous studies; however, back then the maximum was observed at site 3 (km 73.86), in close vicinity to the former chemical plant, with decreasing values in downstream direction. This could indicate a substantial downstream transport of contaminated sediment particles in recent years. In this context, it has to be noted that prior to a state horticultural show in 2002, sediment from the area downstream of the former chemical plant was removed by dredging possibly explaining lower *w*(Hg)_total_ at site 3 (km 73.86). Also, for total Hg, a drop after the confluence of both canals can be seen.

The *w*(Hg) of MMHg and total Hg in samples 5 (km 77.94) and 8 (km 96) might be lower than at neighboring sites (km 75.9 and 80.99, and km 104, respectively) because here the sampling could only be done in small bays that were characterized by very low streaming and considerably less amount of fine-grain sediment than observed at the other sampling sites. Therefore, suspended contaminated sediment transported with the streaming is less likely to be deposited there. However, for a detailed investigation, a representative sampling regarding area and depth would have to be conducted.

The soil sample W, taken at the level of km 79.5, fits in with the overall trend of MMHg and total Hg contamination, with mass fractions of (41 ± 4) μg/kg and (117 ± 3) mg/kg, respectively. This is rather surprising because no further Hg/MMHg deposition could have taken place since Finow Canal sediment was placed there for the last time around 40 years ago. Apparently, the topsoil is still heavily contaminated, and no considerable changes have occurred. Of course, it has to be said that this statement is based on only one sample from one location, and *w*(Hg)_total_ and *w*(Hg)_MMHg_ may vary considerably in the whole washing bed due to variations in the soil conditions. Because this is not the only washing bed where sediment from Finow Canal has been deposited in the past, it is to be expected that these washing beds contaminated with legacy Hg have been and will be contributing largely to atmospheric Hg^0^ emissions, as soils have been described as a source for Hg^0^ [22].

**Fig. 5:**
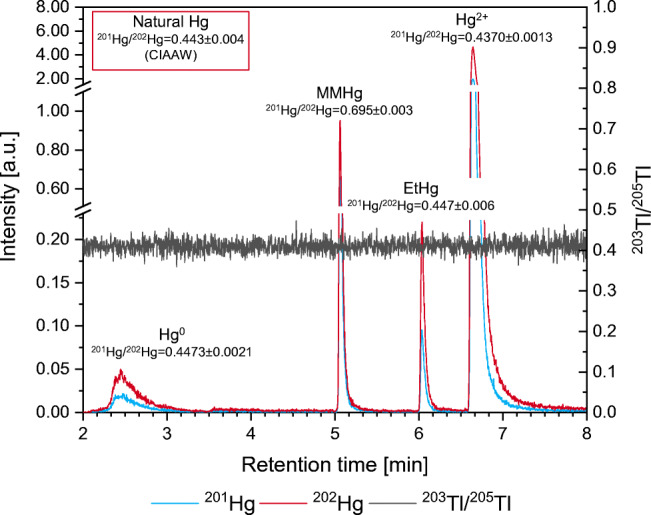
Exemplary Hg speciation results via GC/ICP-ToF-MS of the soil sample (sample W) spiked with ^201^Hg-enriched MMHg. Annotated ^201^Hg/^202^Hg isotope ratios and uncertainties were determined from *m* = 3 measurements, and the natural isotope ratio was calculated from isotopic abundances and corresponding uncertainties taken from CIAAW. The ^201^Hg/^202^Hg of all species but MMHg are similar to the natural isotope ratio indicating that no species transformation takes place during sample preparation and analysis

The chromatogram of the soil sample (Fig. 5) shows the Hg speciation which was similar for all samples. The four species that could be identified by their retention time were elemental Hg (Hg^0^), MMHg, monoethyl-Hg (EtHg), and inorganic Hg (Hg^2+^), detected as Hg^0^, methylpropyl-Hg, ethylpropyl-Hg, and dipropyl-Hg, respectively. Several small peaks could be observed on the tailing of Hg^2+^, but they could not be identified and are probably artifacts produced during derivatization. It was shown in previous works that Hg^0^ is produced by reduction of analytes during sample preparation and/or analysis [44], whereas the amount of Hg^0^ present in the sample is lost during freeze-drying. Since it is described as rather unstable [24], EtHg most probably originates from the sample and is not an artifact, but the amount of EtHg indicated by the peak size seems to be surprisingly high. Unfortunately, due to the noted instability, quantification of EtHg is difficult, which is also highlighted by the lack of appropriate reference materials.

To investigate and exclude potential species transformation, the sample was spiked with ^201^Hg-enriched MMHg. Comparing the ^201^Hg/^202^Hg of all species with the natural isotope ratio, it can be seen that all species but MMHg show an isotope ratio similar to the natural one. This indicates that no significant demethylation or reduction of MMHg takes place during sample preparation and analysis. Otherwise, the ^201^Hg/^202^Hg would have been shifted to values higher than natural.

The amount of substance fraction of MMHg/total Hg of all samples was between 0.029 and 0.32% corresponding to the proposed natural fraction of 0.1–1% [23]. No trends were observed between samples with supposedly natural *w*(Hg)_total_ and *w*(Hg)_MMHg_ (samples 1 and 2) and samples affected by industrial contamination.

## Conclusions

The performance of ICP-ToF-MS for SSID GC/ICP-MS analysis of MMHg was compared with ICP-Q-MS and ICP-SF-MS. As opposed to the use in continuous liquid introduction, no improvement of isotope ratio precision was observed by using the ToF mass analyzer for transient signals when investigating only a limited number of isotopes. However, the (quasi-) simultaneous detection of the whole mass spectrum will probably lead to much better results of ICP-ToF-MS compared to the other single-collector ICP-MS instruments, when more than one isotope system is used. This could be investigated further, e.g., by additionally analyzing organotin and organolead species, which can also be derivatized using aqueous phase alkylation to be accessible for GC speciation [13, 14].

Furthermore, the SSID GC/ICP-ToF-MS method was applied for the analysis of MMHg in sediments of Finow Canal, which is a heavily industrially polluted canal in Northern Germany. The high *w*(Hg)_MMHg_ determined in the samples show that natural (bio-)methylation of Hg is not hindered by the partially extremely high Hg concentration in the sediment. The analysis of one topsoil sample from a washing bed where contaminated sediment had been deposited around 40 years ago shows the same high values of both, *w*(Hg)_total_ and *w*(Hg)_MMHg_. This means that Finow Canal sediments will act as a source for MMHg in this ecosystem for long time, whereas the sediment removed from the canal and deposited on washing beds contributes to atmospheric Hg^0^ emissions. It might be interesting to analyze also other environmental compartments (water, fish, plants) of this region to assess the overall status of MMHg distribution. Also, further investigation on the occurrence of EtHg could be interesting to see if it is produced within the sediment/soil or if it was released from the chemical plant because EtHg has been used as a component of seed dressings.

## Supplementary Information

ESM 1 (PDF 56 kb)
