# Supplementary material for: Species-specific isotope dilution analysis of monomethylmercury in sediment using GC/ICP-ToF-MS and comparison with ICP-Q-MS and ICP-SF-MS
Source: Anal Bioanal Chem. 2021 Jul 23;413(21):5279–89. doi: 10.1007/s00216-021-03497-z (PMC8405517; doi:10.1007/s00216-021-03497-z)
Supplement: Supplementary file 1 — (PDF 56 kb) [file 216_2021_3497_MOESM1_ESM.pdf]

<u>Supplementary Information</u>

Species-specific isotope dilution analysis of monomethylmercury in sediment using GC/ICP-ToF-MS and comparison with ICP-Q-MS and ICP-SF-MS

Sebastian Faßbender, Marcus von der Au, Maren Koenig, Jürgen Pelzer, Christian Piechotta, Jochen Vogl, Björn Meermann

Table of contents:

S1	Preparation of samples for determination of MMHg in sediment
Table S1	Details of the sampling sites
Table S2	Details of the ^{201}Hg-enriched spike and reference materials
Table S3	Operating conditions of the GC/ICP-MS interface for MMHg analysis
Table S4	Operating conditions of the ICP-ToF-MS instrument for MMHg analysis
Table S5	Operating conditions of the ICP-Q-MS instrument for MMHg analysis
Table S6	Operating conditions of the ICP-SF-MS instrument for MMHg analysis
Table S7	Operating conditions of the ICP-SF-MS instrument for total Hg analysis
Table S8	MMHg ($w(Hg)_{MMHg}$) and Hg ($w(Hg)_{total}$) mass fractions determined in sediment reference materials and samples with expanded uncertainty U (k = 2) based on dry weight and n = 3 replicates (exceptions annotated)

S1 Preparation of samples for determination of MMHg in sediment.

A sample mass of 250 mg was weighed into a 15 mL centrifuge tube. Then, 1 mL of aqueous $CuSO_4$ solution (1 mol/L) and the ^{201}Hg-enriched MMHg spike solution were added. The required volume of spike solution was estimated for every sample to target an $^{201}Hg/^{202}Hg$ ratio of approximately 1 and the actual amount of spike solution added was determined gravimetrically. To assess the required volume of spike solution, the MMHg concentration of the samples had been screened with an external calibration technique beforehand. The sample-spike mixture was vortexed thoroughly to ensure complete mixing. A volume of 4 mL HNO_3 (3 mol/L) and 5 mL of DCM were added for simultaneous extraction of MMHg from the sediment and liquid-liquid extraction into DCM for separation from inorganic Hg. The tube was vortexed in the dark for 30 min and subsequently centrifuged for 30 min at 3000 min^{-1}. The DCM phase was separated over a phase separation filter and mixed with 4.5 mL of aqueous $Na_2S_2O_3$ solution (0.1 mmol/L) to back extract MMHg into the aqueous phase for derivatization. The tube was shaken for 30 min at 300 min^{-1} on a horizontal shaker and subsequently centrifuged for 10 min at 3000 min^{-1}. A volume of 4 mL of the top aqueous phase was transferred into a new 50 mL centrifuge tube. For derivatization, 19 mL of a buffer mixture containing glacial acetic acid, methanol, and ultrapure water in a ratio of 1:1:1.5 adjusted to pH 4.5 with NaOH solution (0.32 kg/kg) was added to the MMHg extract. Then, 1 mL of a solution of the derivatization agent $NaBPr_4$ in THF (20 g/L) and 1 mL of n-hexane for extraction of the derivatized MMHg were added. The derivatization took place during 1 h of shaking at 240 min^{-1} on a horizontal shaker. Afterwards, the tube was centrifuged for 10 min at 3500 min^{-1}, and the hexane phase was transferred into a 2 mL microcentrifuge tube. The derivatization procedure was repeated by adding another 1 mL of $NaBPr_4$ solution in THF (20 g/L) and 1 mL of n-hexane to the 50 mL centrifuge tube. After shaking and centrifuging, the hexane phase was unified with the first one in the microcentrifuge tube. The tube was vortexed, and anhydrous Na_2SO_4 was added to bind traces of residual water. Finally, 1 mL of the sample solution was transferred into a 2 mL amber glass GC vial for analysis. Very low concentrated sediment samples (IAEA-456 and samples 1 and 8) were prepared with a sample amount of 1 g and the twofold volume of reagents in the extraction procedure. All other steps were the same as for higher concentrated samples. Procedural blanks were prepared by following the whole sample preparation procedure without sediment and with (for determination of LOQ) and without ^{201}Hg-enriched MMHg spike.

Table S1 Details of the sampling site

Sample	Material	Chainage km	Coordinates
1	Sediment	57.4	52°50'45.1"N 13°32'32.1"E
2	Sediment	62	52°50'21.7"N 13°36'10.5"E
3	Sediment	73.86	52°50'35.2"N 13°46'10.0"E
4	Sediment	75.9	52°50'19.3"N 13°47'31.6"E
5	Sediment	77.94	52°50'10.5"N 13°49'15.0"E
W	Soil	79.5	52°50'42.5"N 13°50'23.2"E
6	Sediment	80.99	52°50'57.8"N 13°51'21.0"E
7	Sediment	88.91	52°50'55.1"N 13°57'09.7"E
8	Sediment	96	52°51'51.2"N 14°02'56.8"E
9	Sediment	104	52°52'30.4"N 14°08'55.3"E

Table S2 Details of the ^{201}Hg-enriched spike and reference materials

Material	MMHg mass fraction (mg/kg)	Total Hg mass fraction (mg/kg)	Isotopic composition
MMHg spike for SSID (MMHg in 3:1 acetic acid/methanol)	5.1 ± 0.3 (as MMHg)	/	^{196}Hg: < 0.1 %<br>^{198}Hg: (0.10 ± 0.03) %<br>^{199}Hg: (0.41 ± 0.06) %<br>^{200}Hg: (2.29 ± 0.05) %<br>^{201}Hg: (94.7 ± 0.1) %<br>^{202}Hg: (2.32 ± 0.06) %<br>^{204}Hg: (0.11 ± 0.02) %
Hg spike for ID (solid HgO)	/	0.92×10^{6}	^{196}Hg: < 0.05 %<br>^{198}Hg: (0.08 ± 0.010) %<br>^{199}Hg: (0.10 ± 0.010) %<br>^{200}Hg: (0.45 ± 0.010) %<br>^{201}Hg: (98.11 ± 0.030) %<br>^{202}Hg: (1.18 ± 0.02) %<br>^{204}Hg: (0.08 ± 0.010) %
IAEA-456 (marine sediment)	$0.0125\times10^{-3} \pm 0.019\times10^{-3}$ (as Hg)	0.077 ± 0.005	/
ERM-CC020 (river sediment)	/	27.4 ± 0.6	/
ERM-CC580 (estuarine sediment)	0.0755 ± 0.0037 (as MMHg)	132 ± 3	/

Table S3 Operating conditions of the GC/ICP-MS interface for MMHg analysis

Parameter	Value
Column	TG5-SilMS (30 m x 0.25 mm i.d., 0.25 µm)
PTV Injector temperature program	250 °C to 400 °C with 10 °C/s
Injection volume (µL)	2
Carrier gas	Helium
Carrier gas flow rate (mL/min)	1
Oven temperature program	1 min hold 40 °C, 20 °C/min to 150 °C,<br>70 °C/min to 300 °C, 4.5 min hold 300 °C
Transfer line temperature (°C)	300
Make up gas flow rate (L/min)	0.600

Table S4 Operating conditions of the ICP-ToF-MS instrument for MMHg analysis

Parameter	Value
Cones	Platinum
RF power (W)	1200
Cool gas flow rate (L/min)	14
Auxiliary gas flow rate (L/min)	0.80
Nebulizer gas flow rate (L/min)	0.730
Integration time (s)	0.2
Isotopes	^{201}Hg, ^{202}Hg, ^{203}Tl, ^{205}Tl

Table S5 Operating conditions of the ICP-Q-MS instrument for MMHg analysis

Parameter	Value
Cones	Nickel
RF power (W)	1550
Cool gas flow rate (L/min)	14
Auxiliary gas flow rate (L/min)	0.80
Nebulizer gas flow rate (L/min)	0.730
Dwell time (s)	0.1
Isotopes	^{201}Hg, ^{202}Hg, ^{203}Tl, ^{205}Tl

Table S6 Operating conditions of the ICP-SF-MS instrument for MMHg analysis

Parameter	Value
Cones	Nickel
RF power (W)	1250
Cool gas flow rate (L/min)	16
Auxiliary gas flow rate (L/min)	0.85
Nebulizer gas flow rate (L/min)	0.530
Resolution	Low (300)
Mass window	20
Samples per peak	100
Sample time (s)	0.02
Isotopes	^{201}Hg, ^{202}Hg, ^{203}Tl, ^{205}Tl

Table S7 Operating conditions of the ICP-SF-MS instrument for total Hg analysis

Parameter	Value
Cones	Nickel
RF power (W)	1250
Cool gas flow rate (L/min)	16
Auxiliary gas flow rate (L/min)	1.00
Nebulizer gas flow rate (L/min)	1.160
Resolution	Low (300)
Mass window	5
Samples per peak	200
Dwell time (s)	0.002
Runs	10
Passes	500
Isotopes	^{200}Hg, ^{201}Hg, ^{202}Hg

Table S8 MMHg ($w(\mathrm{Hg})_{\mathrm{MMHg}}$) and Hg ($w(\mathrm{Hg})_{\mathrm{total}}$) mass fractions determined in sediment reference materials and samples with expanded uncertainty U (k = 2) based on dry weight and n = 3 replicates (exceptions annotated)

**Sample**	**MMHg mass fraction (µg/kg)**	**Total Hg mass fraction (mg/kg)**
ERM-CC020		27.5 ± 1.0
ERM-CC020 certified value		27.4 ± 0.6
ERM-CC580	81.8 ± 5.3[a]<br>82.3 ± 4.2[b]<br>81.8 ± 4.2[c]	136.5 ± 4.5[d]
ERM-CC580 certified value	75.5 ± 3.7	132 ± 3
IAEA-456	0.132 ± 0.013[e]	0.0823 ± 0.0070
IAEA-456 certified value	0.125 ± 0.019	0.077 ± 0.005
Sediment 1 (km 57.4)	0.180 ± 0.017	0.056 ± 0.013
Sediment 2 (km 62)	0.488 ± 0.043	0.259 ± 0.012
Sediment 3 (km 73.86)	26.3 ± 1.7	9.14 ± 0.27
Sediment 4 (km 75.9)	39.3 ± 5.5	41.4 ± 2.6
Sediment 5 (km 77.94)	20.5 ± 1.8	9.18 ± 0.69
Sediment 6 (km 80.99)	36.3 ± 2.7	126.1 ± 7.1
Sediment 7 (km 88.91)	33.0 ± 3.5	30.0 ± 4.2
Sediment 8 (km 96)	0.367 ± 0.033	0.549 ± 0.066
Sediment 9 (km 104)	6.37 ± 0.54	2.58 ± 0.12
Washing bed (W)	40.6 ± 3.7	117.2 ± 3.3

[a] as MMHg (w(MMHg)), n = 12, determined with GC/ICP-ToF-MS
[b] as MMHg (w(MMHg)), n = 2, determined with GC/ICP-Q-MS
[c] as MMHg (w(MMHg)), n = 2, determined with GC/ICP-SF-MS
[d] as MMHg (w(MMHg))
[e] n = 6
